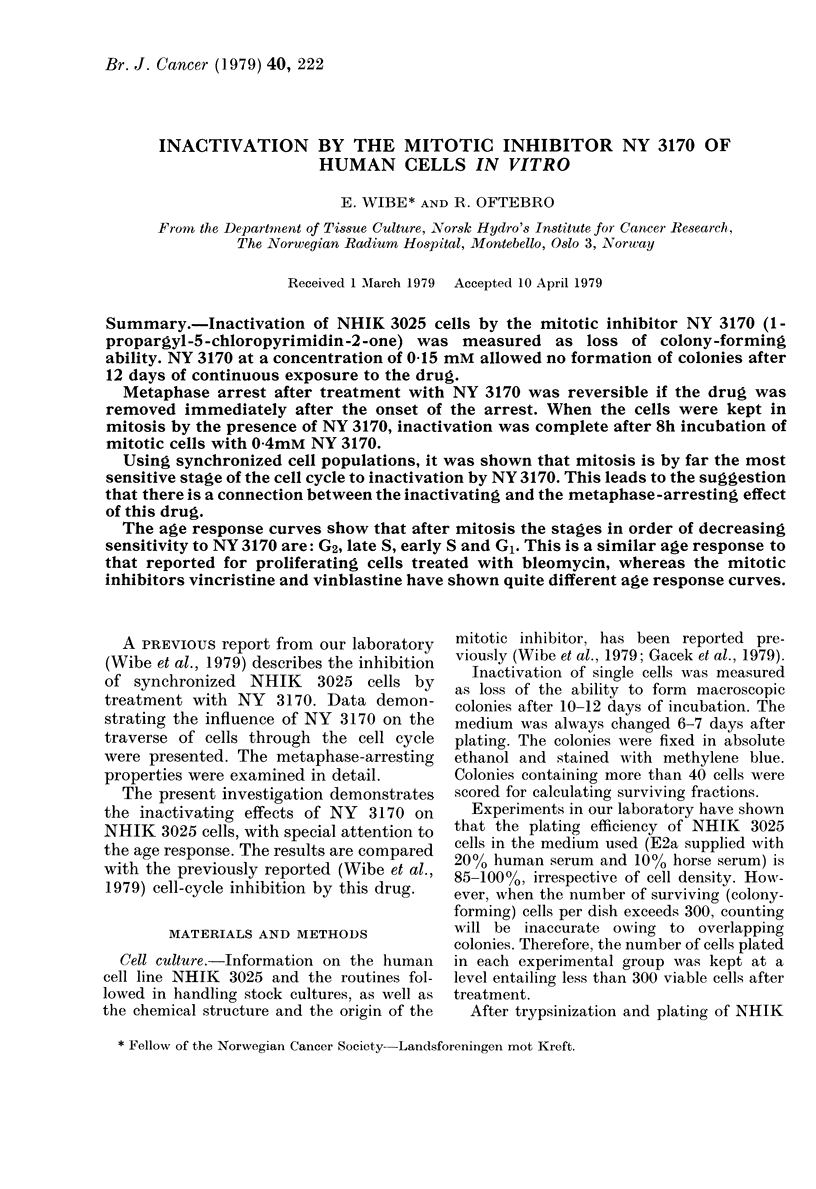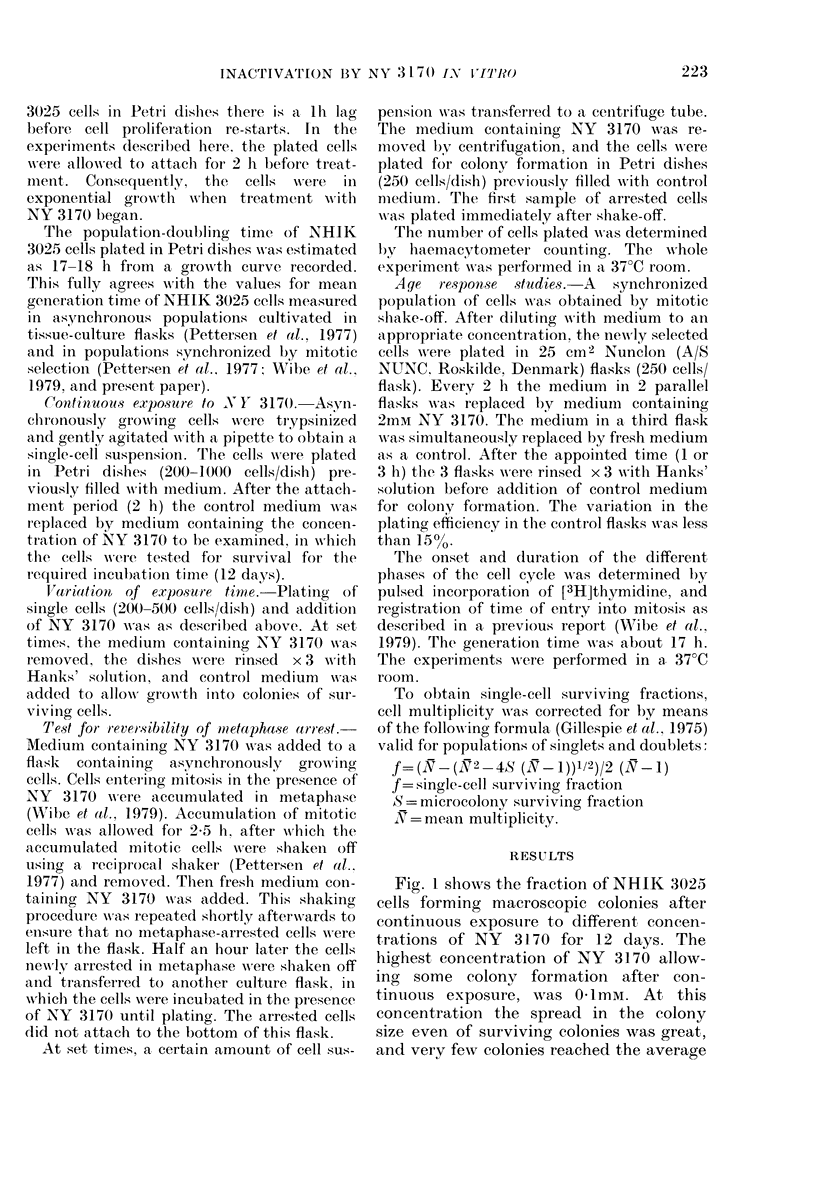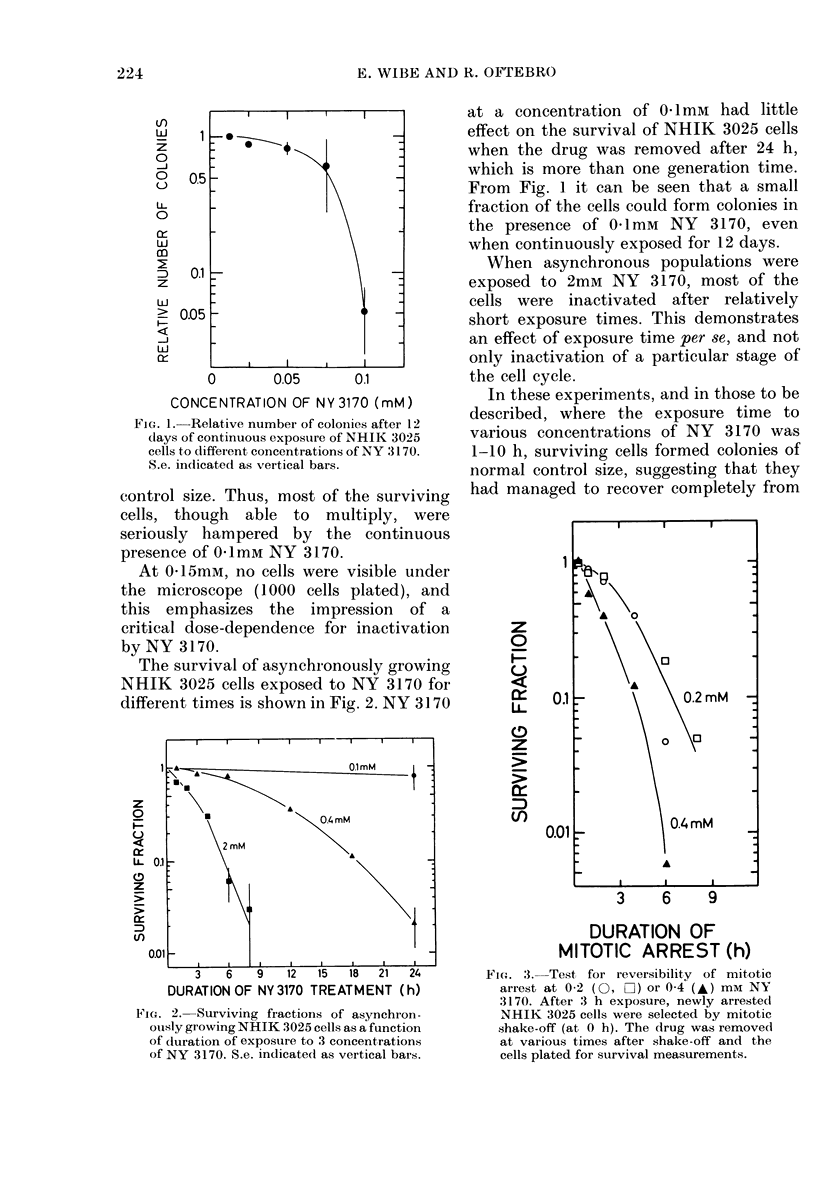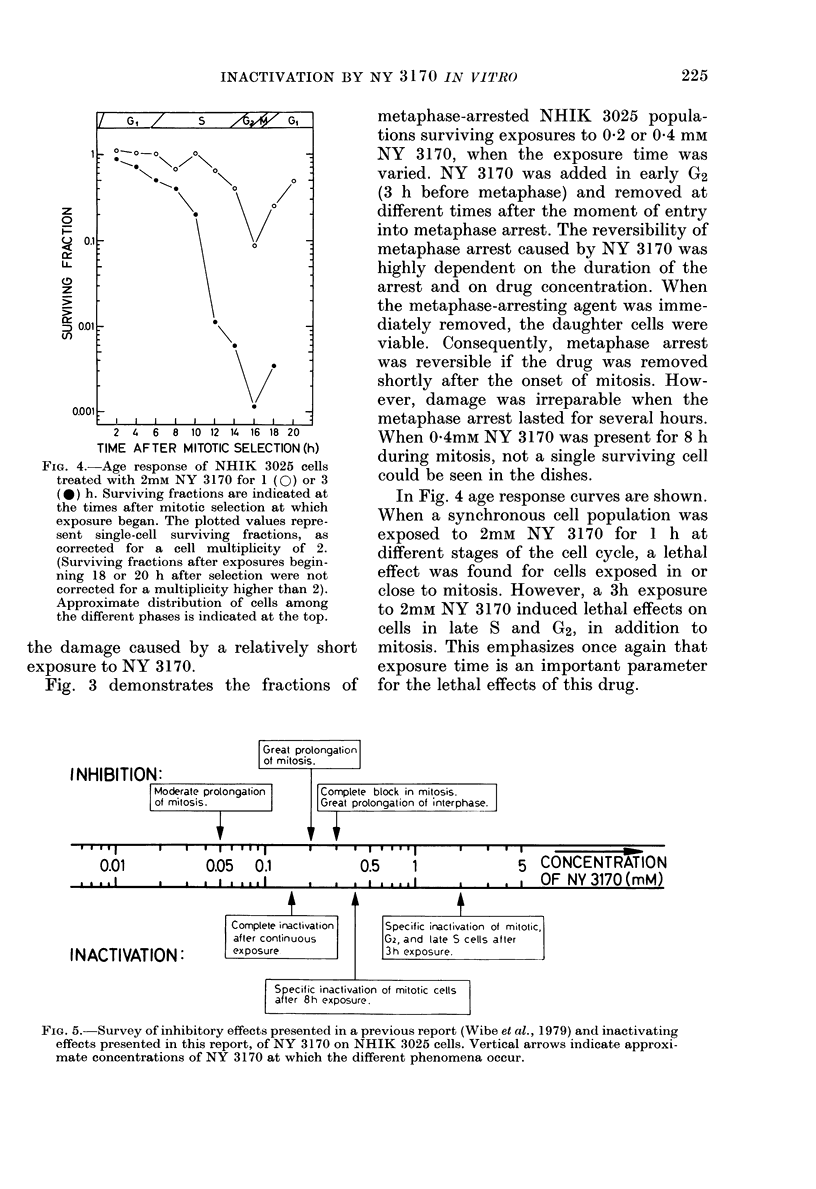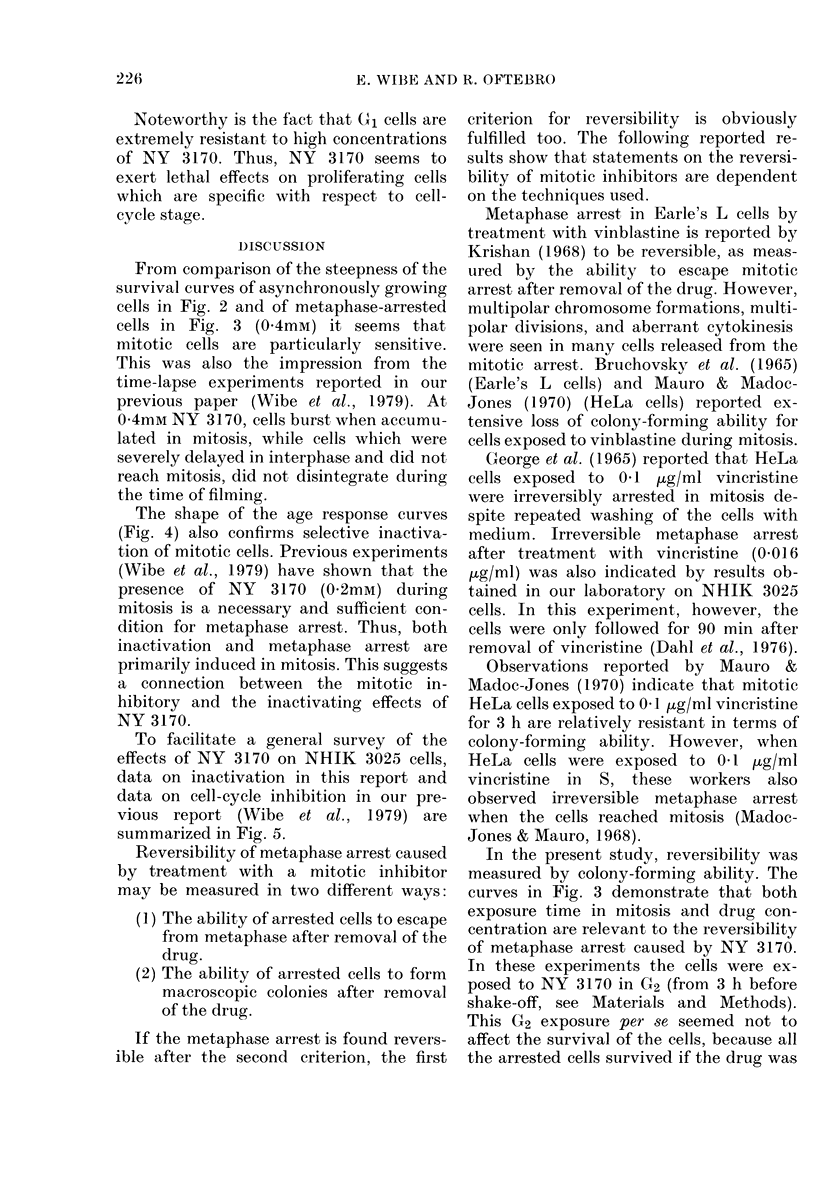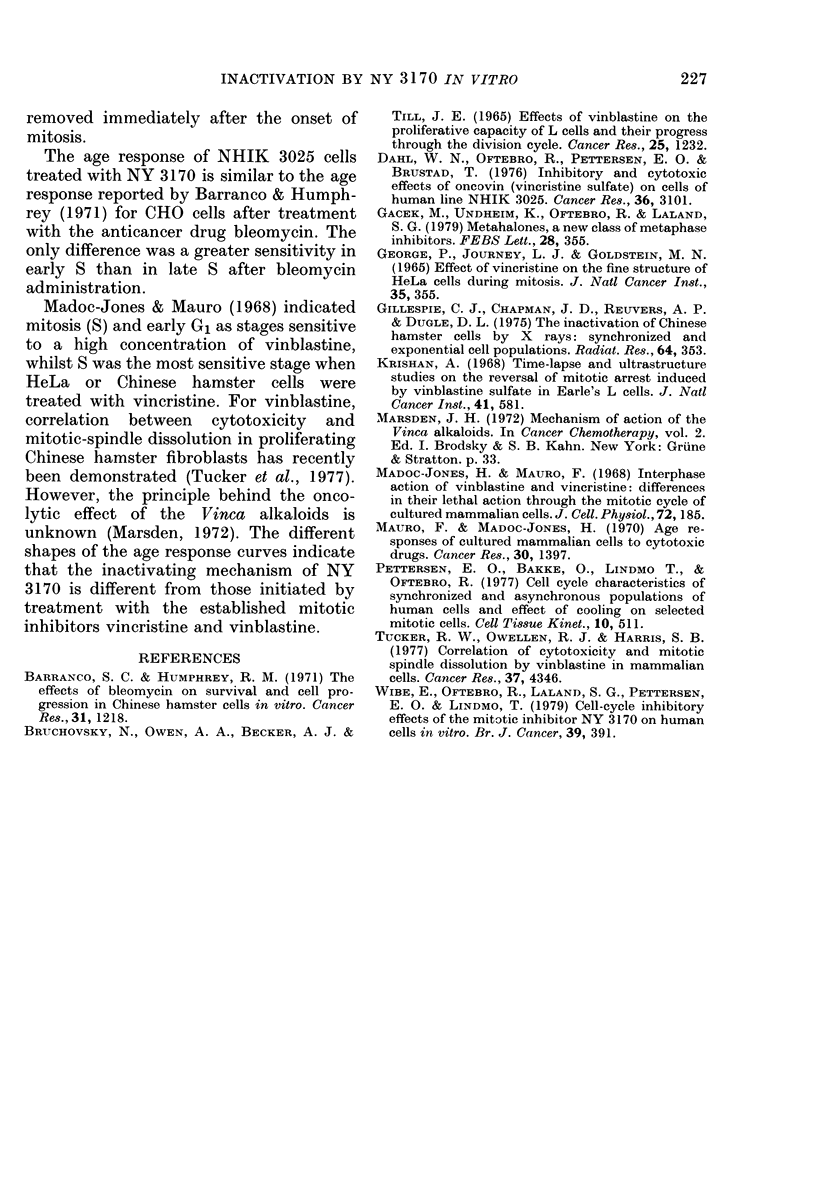# Inactivation by the mitotic inhibitor NY 3170 of human cells in vitro.

**DOI:** 10.1038/bjc.1979.169

**Published:** 1979-08

**Authors:** E. Wibe, R. Oftebro

## Abstract

Inactivation of NHIK 3025 cells ny the mitotic inhibitor NY 3170 (1-propargyl-5-chloropyrimidin-2-one) was measured as loss of colony-forming ability. NY 3170 at a concentration of 0.15 nM allowed no formation of colonies after 12 days of continuous exposure to the drug. Metaphase arrest after treatment with NY 3170 was reversible if the drug was removed immediately after the onset of the arrest. When the cells were kept in mitosis by the presence of NY 3170, inactivation was complete after 8h incubation of mitotic cells with 0.4 nM NY 3170. Using synchronized cell populations, it was shown that mitosis is by far the most sensitive stage of the cell cycle to inactivation by NY 3170. This leads to the suggestion that there is a connection between the inactivating and the metaphase-arresting effect of this drug. The age response curves show that after mitosis the stages in order of decreasing sensitivity to NY3170 are: G2, late S, early S and G1. This is a similar age response to that reported for proliferating cells treated with bleomycin, whereas the mitotic inhibitors vincristine and vinblastine have shown qhite different age response curves.


Br. J. Cancer (1979) 40, 222

### INACTIVATION BY THE MITOTIC INHIBITOR NY 3170 OF HUMAN CELLS *IN VITRO*

E. WIBE* AND R. OFTEBRO

*From the Department of Tissue Culture, Norsk Hydro's Institute for Cancer Research, The Norwegian Radium Hospital, Montebello, Oslo 3, Norway*

Received 1 March 1979 Accepted 10 April 1979

**Summary.—Inactivation of NHIK 3025 cells by the mitotic inhibitor NY 3170 (1-propargyl-5-chloropyrimidin-2-one) was measured as loss of colony-forming ability. NY 3170 at a concentration of 0·15 mM allowed no formation of colonies after 12 days of continuous exposure to the drug.**

**Metaphase arrest after treatment with NY 3170 was reversible if the drug was removed immediately after the onset of the arrest. When the cells were kept in mitosis by the presence of NY 3170, inactivation was complete after 8h incubation of mitotic cells with 0·4mM NY 3170.**

**Using synchronized cell populations, it was shown that mitosis is by far the most sensitive stage of the cell cycle to inactivation by NY 3170. This leads to the suggestion that there is a connection between the inactivating and the metaphase-arresting effect of this drug.**

**The age response curves show that after mitosis the stages in order of decreasing sensitivity to NY 3170 are: $G_2$, late S, early S and $G_1$. This is a similar age response to that reported for proliferating cells treated with bleomycin, whereas the mitotic inhibitors vincristine and vinblastine have shown quite different age response curves.**

A PREVIOUS report from our laboratory (Wibe *et al.*, 1979) describes the inhibition of synchronized NHIK 3025 cells by treatment with NY 3170. Data demonstrating the influence of NY 3170 on the traverse of cells through the cell cycle were presented. The metaphase-arresting properties were examined in detail.

The present investigation demonstrates the inactivating effects of NY 3170 on NHIK 3025 cells, with special attention to the age response. The results are compared with the previously reported (Wibe *et al.*, 1979) cell-cycle inhibition by this drug.

### MATERIALS AND METHODS

*Cell culture.*—Information on the human cell line NHIK 3025 and the routines followed in handling stock cultures, as well as the chemical structure and the origin of the mitotic inhibitor, has been reported previously (Wibe *et al.*, 1979; Gacek *et al.*, 1979).

Inactivation of single cells was measured as loss of the ability to form macroscopic colonies after 10–12 days of incubation. The medium was always changed 6–7 days after plating. The colonies were fixed in absolute ethanol and stained with methylene blue. Colonies containing more than 40 cells were scored for calculating surviving fractions.

Experiments in our laboratory have shown that the plating efficiency of NHIK 3025 cells in the medium used (E2a supplied with 20% human serum and 10% horse serum) is 85–100%, irrespective of cell density. However, when the number of surviving (colony-forming) cells per dish exceeds 300, counting will be inaccurate owing to overlapping colonies. Therefore, the number of cells plated in each experimental group was kept at a level entailing less than 300 viable cells after treatment.

After trypsinization and plating of NHIK

* Fellow of the Norwegian Cancer Society—Landsforeningen mot Kreft.

3025 cells in Petri dishes there is a 1h lag before cell proliferation re-starts. In the experiments described here, the plated cells were allowed to attach for 2 h before treatment. Consequently, the cells were in exponential growth when treatment with NY 3170 began.

The population-doubling time of NHIK 3025 cells plated in Petri dishes was estimated as 17–18 h from a growth curve recorded. This fully agrees with the values for mean generation time of NHIK 3025 cells measured in asynchronous populations cultivated in tissue-culture flasks (Pettersen *et al.*, 1977) and in populations synchronized by mitotic selection (Pettersen *et al.*, 1977; Wibe *et al.*, 1979, and present paper).

*Continuous exposure to NY 3170.*—Asynchronously growing cells were trypsinized and gently agitated with a pipette to obtain a single-cell suspension. The cells were plated in Petri dishes (200–1000 cells/dish) previously filled with medium. After the attachment period (2 h) the control medium was replaced by medium containing the concentration of NY 3170 to be examined, in which the cells were tested for survival for the required incubation time (12 days).

*Variation of exposure time.*—Plating of single cells (200–500 cells/dish) and addition of NY 3170 was as described above. At set times, the medium containing NY 3170 was removed, the dishes were rinsed ×3 with Hanks' solution, and control medium was added to allow growth into colonies of surviving cells.

*Test for reversibility of metaphase arrest.*—Medium containing NY 3170 was added to a flask containing asynchronously growing cells. Cells entering mitosis in the presence of NY 3170 were accumulated in metaphase (Wibe *et al.*, 1979). Accumulation of mitotic cells was allowed for 2·5 h, after which the accumulated mitotic cells were shaken off using a reciprocal shaker (Pettersen *et al.*, 1977) and removed. Then fresh medium containing NY 3170 was added. This shaking procedure was repeated shortly afterwards to ensure that no metaphase-arrested cells were left in the flask. Half an hour later the cells newly arrested in metaphase were shaken off and transferred to another culture flask, in which the cells were incubated in the presence of NY 3170 until plating. The arrested cells did not attach to the bottom of this flask.

At set times, a certain amount of cell suspension was transferred to a centrifuge tube. The medium containing NY 3170 was removed by centrifugation, and the cells were plated for colony formation in Petri dishes (250 cells/dish) previously filled with control medium. The first sample of arrested cells was plated immediately after shake-off.

The number of cells plated was determined by haemacytometer counting. The whole experiment was performed in a 37°C room.

*Age response studies.*—A synchronized population of cells was obtained by mitotic shake-off. After diluting with medium to an appropriate concentration, the newly selected cells were plated in 25 cm² Nunclon (A/S NUNC, Roskilde, Denmark) flasks (250 cells/flask). Every 2 h the medium in 2 parallel flasks was replaced by medium containing 2mM NY 3170. The medium in a third flask was simultaneously replaced by fresh medium as a control. After the appointed time (1 or 3 h) the 3 flasks were rinsed ×3 with Hanks' solution before addition of control medium for colony formation. The variation in the plating efficiency in the control flasks was less than 15%.

The onset and duration of the different phases of the cell cycle was determined by pulsed incorporation of [$^3$H]thymidine, and registration of time of entry into mitosis as described in a previous report (Wibe *et al.*, 1979). The generation time was about 17 h. The experiments were performed in a 37°C room.

To obtain single-cell surviving fractions, cell multiplicity was corrected for by means of the following formula (Gillespie *et al.*, 1975) valid for populations of singlets and doublets:

$$f = (\bar{N} - (\bar{N}^2 - 4S\ (\bar{N}-1))^{1/2})/2\ (\bar{N}-1)$$

$f$ = single-cell surviving fraction
$S$ = microcolony surviving fraction
$\bar{N}$ = mean multiplicity.

### RESULTS

Fig. 1 shows the fraction of NHIK 3025 cells forming macroscopic colonies after continuous exposure to different concentrations of NY 3170 for 12 days. The highest concentration of NY 3170 allowing some colony formation after continuous exposure, was 0·1mM. At this concentration the spread in the colony size even of surviving colonies was great, and very few colonies reached the average

Fig. 1.—Relative number of colonies after 12 days of continuous exposure of NHIK 3025 cells to different concentrations of NY 3170. S.e. indicated as vertical bars.

control size. Thus, most of the surviving cells, though able to multiply, were seriously hampered by the continuous presence of 0·1mM NY 3170.

At 0·15mM, no cells were visible under the microscope (1000 cells plated), and this emphasizes the impression of a critical dose-dependence for inactivation by NY 3170.

The survival of asynchronously growing NHIK 3025 cells exposed to NY 3170 for different times is shown in Fig. 2. NY 3170

Fig. 2.—Surviving fractions of asynchronously growing NHIK 3025 cells as a function of duration of exposure to 3 concentrations of NY 3170. S.e. indicated as vertical bars.

at a concentration of 0·1mM had little effect on the survival of NHIK 3025 cells when the drug was removed after 24 h, which is more than one generation time. From Fig. 1 it can be seen that a small fraction of the cells could form colonies in the presence of 0·1mM NY 3170, even when continuously exposed for 12 days.

When asynchronous populations were exposed to 2mM NY 3170, most of the cells were inactivated after relatively short exposure times. This demonstrates an effect of exposure time *per se*, and not only inactivation of a particular stage of the cell cycle.

In these experiments, and in those to be described, where the exposure time to various concentrations of NY 3170 was 1–10 h, surviving cells formed colonies of normal control size, suggesting that they had managed to recover completely from

Fig. 3.—Test for reversibility of mitotic arrest at 0·2 (○, □) or 0·4 (▲) mM NY 3170. After 3 h exposure, newly arrested NHIK 3025 cells were selected by mitotic shake-off (at 0 h). The drug was removed at various times after shake-off and the cells plated for survival measurements.

FIG. 4.—Age response of NHIK 3025 cells treated with 2mM NY 3170 for 1 (○) or 3 (●) h. Surviving fractions are indicated at the times after mitotic selection at which exposure began. The plotted values represent single-cell surviving fractions, as corrected for a cell multiplicity of 2. (Surviving fractions after exposures beginning 18 or 20 h after selection were not corrected for a multiplicity higher than 2). Approximate distribution of cells among the different phases is indicated at the top.

the damage caused by a relatively short exposure to NY 3170.

Fig. 3 demonstrates the fractions of metaphase-arrested NHIK 3025 populations surviving exposures to 0·2 or 0·4 mM NY 3170, when the exposure time was varied. NY 3170 was added in early $G_2$ (3 h before metaphase) and removed at different times after the moment of entry into metaphase arrest. The reversibility of metaphase arrest caused by NY 3170 was highly dependent on the duration of the arrest and on drug concentration. When the metaphase-arresting agent was immediately removed, the daughter cells were viable. Consequently, metaphase arrest was reversible if the drug was removed shortly after the onset of mitosis. However, damage was irreparable when the metaphase arrest lasted for several hours. When 0·4mM NY 3170 was present for 8 h during mitosis, not a single surviving cell could be seen in the dishes.

In Fig. 4 age response curves are shown. When a synchronous cell population was exposed to 2mM NY 3170 for 1 h at different stages of the cell cycle, a lethal effect was found for cells exposed in or close to mitosis. However, a 3h exposure to 2mM NY 3170 induced lethal effects on cells in late S and $G_2$, in addition to mitosis. This emphasizes once again that exposure time is an important parameter for the lethal effects of this drug.

FIG. 5.—Survey of inhibitory effects presented in a previous report (Wibe *et al.*, 1979) and inactivating effects presented in this report, of NY 3170 on NHIK 3025 cells. Vertical arrows indicate approximate concentrations of NY 3170 at which the different phenomena occur.

Noteworthy is the fact that $G_1$ cells are extremely resistant to high concentrations of NY 3170. Thus, NY 3170 seems to exert lethal effects on proliferating cells which are specific with respect to cell-cycle stage.

### DISCUSSION

From comparison of the steepness of the survival curves of asynchronously growing cells in Fig. 2 and of metaphase-arrested cells in Fig. 3 (0·4mM) it seems that mitotic cells are particularly sensitive. This was also the impression from the time-lapse experiments reported in our previous paper (Wibe *et al.*, 1979). At 0·4mM NY 3170, cells burst when accumulated in mitosis, while cells which were severely delayed in interphase and did not reach mitosis, did not disintegrate during the time of filming.

The shape of the age response curves (Fig. 4) also confirms selective inactivation of mitotic cells. Previous experiments (Wibe *et al.*, 1979) have shown that the presence of NY 3170 (0·2mM) during mitosis is a necessary and sufficient condition for metaphase arrest. Thus, both inactivation and metaphase arrest are primarily induced in mitosis. This suggests a connection between the mitotic inhibitory and the inactivating effects of NY 3170.

To facilitate a general survey of the effects of NY 3170 on NHIK 3025 cells, data on inactivation in this report and data on cell-cycle inhibition in our previous report (Wibe *et al.*, 1979) are summarized in Fig. 5.

Reversibility of metaphase arrest caused by treatment with a mitotic inhibitor may be measured in two different ways:

(1) The ability of arrested cells to escape from metaphase after removal of the drug.
(2) The ability of arrested cells to form macroscopic colonies after removal of the drug.

If the metaphase arrest is found reversible after the second criterion, the first criterion for reversibility is obviously fulfilled too. The following reported results show that statements on the reversibility of mitotic inhibitors are dependent on the techniques used.

Metaphase arrest in Earle's L cells by treatment with vinblastine is reported by Krishan (1968) to be reversible, as measured by the ability to escape mitotic arrest after removal of the drug. However, multipolar chromosome formations, multipolar divisions, and aberrant cytokinesis were seen in many cells released from the mitotic arrest. Bruchovsky *et al.* (1965) (Earle's L cells) and Mauro & Madoc-Jones (1970) (HeLa cells) reported extensive loss of colony-forming ability for cells exposed to vinblastine during mitosis.

George *et al.* (1965) reported that HeLa cells exposed to 0·1 $\mu$g/ml vincristine were irreversibly arrested in mitosis despite repeated washing of the cells with medium. Irreversible metaphase arrest after treatment with vincristine (0·016 $\mu$g/ml) was also indicated by results obtained in our laboratory on NHIK 3025 cells. In this experiment, however, the cells were only followed for 90 min after removal of vincristine (Dahl *et al.*, 1976).

Observations reported by Mauro & Madoc-Jones (1970) indicate that mitotic HeLa cells exposed to 0·1 $\mu$g/ml vincristine for 3 h are relatively resistant in terms of colony-forming ability. However, when HeLa cells were exposed to 0·1 $\mu$g/ml vincristine in S, these workers also observed irreversible metaphase arrest when the cells reached mitosis (Madoc-Jones & Mauro, 1968).

In the present study, reversibility was measured by colony-forming ability. The curves in Fig. 3 demonstrate that both exposure time in mitosis and drug concentration are relevant to the reversibility of metaphase arrest caused by NY 3170. In these experiments the cells were exposed to NY 3170 in $G_2$ (from 3 h before shake-off, see Materials and Methods). This $G_2$ exposure *per se* seemed not to affect the survival of the cells, because all the arrested cells survived if the drug was removed immediately after the onset of mitosis.

The age response of NHIK 3025 cells treated with NY 3170 is similar to the age response reported by Barranco & Humphrey (1971) for CHO cells after treatment with the anticancer drug bleomycin. The only difference was a greater sensitivity in early S than in late S after bleomycin administration.

Madoc-Jones & Mauro (1968) indicated mitosis (S) and early $G_1$ as stages sensitive to a high concentration of vinblastine, whilst S was the most sensitive stage when HeLa or Chinese hamster cells were treated with vincristine. For vinblastine, correlation between cytotoxicity and mitotic-spindle dissolution in proliferating Chinese hamster fibroblasts has recently been demonstrated (Tucker *et al.*, 1977). However, the principle behind the oncolytic effect of the *Vinca* alkaloids is unknown (Marsden, 1972). The different shapes of the age response curves indicate that the inactivating mechanism of NY 3170 is different from those initiated by treatment with the established mitotic inhibitors vincristine and vinblastine.

### REFERENCES

Barranco, S. C. & Humphrey, R. M. (1971) The effects of bleomycin on survival and cell progression in Chinese hamster cells *in vitro*. *Cancer Res.*, **31**, 1218.

Bruchovsky, N., Owen, A. A., Becker, A. J. & Till, J. E. (1965) Effects of vinblastine on the proliferative capacity of L cells and their progress through the division cycle. *Cancer Res.*, **25**, 1232.

Dahl, W. N., Oftebro, R., Pettersen, E. O. & Brustad, T. (1976) Inhibitory and cytotoxic effects of oncovin (vincristine sulfate) on cells of human line NHIK 3025. *Cancer Res.*, **36**, 3101.

Gacek, M., Undheim, K., Oftebro, R. & Laland, S. G. (1979) Metahalones, a new class of metaphase inhibitors. *FEBS Lett.*, **28**, 355.

George, P., Journey, L. J. & Goldstein, M. N. (1965) Effect of vincristine on the fine structure of HeLa cells during mitosis. *J. Natl Cancer Inst.*, **35**, 355.

Gillespie, C. J., Chapman, J. D., Reuvers, A. P. & Dugle, D. L. (1975) The inactivation of Chinese hamster cells by X rays: synchronized and exponential cell populations. *Radiat. Res.*, **64**, 353.

Krishan, A. (1968) Time-lapse and ultrastructure studies on the reversal of mitotic arrest induced by vinblastine sulfate in Earle's L cells. *J. Natl Cancer Inst.*, **41**, 581.

Marsden, J. H. (1972) Mechanism of action of the *Vinca* alkaloids. In *Cancer Chemotherapy*, vol. 2. Ed. I. Brodsky & S. B. Kahn. New York: Grüne & Stratton. p. 33.

Madoc-Jones, H. & Mauro, F. (1968) Interphase action of vinblastine and vincristine: differences in their lethal action through the mitotic cycle of cultured mammalian cells. *J. Cell. Physiol.*, **72**, 185.

Mauro, F. & Madoc-Jones, H. (1970) Age responses of cultured mammalian cells to cytotoxic drugs. *Cancer Res.*, **30**, 1397.

Pettersen, E. O., Bakke, O., Lindmo T., & Oftebro, R. (1977) Cell cycle characteristics of synchronized and asynchronous populations of human cells and effect of cooling on selected mitotic cells. *Cell Tissue Kinet.*, **10**, 511.

Tucker, R. W., Owellen, R. J. & Harris, S. B. (1977) Correlation of cytotoxicity and mitotic spindle dissolution by vinblastine in mammalian cells. *Cancer Res.*, **37**, 4346.

Wibe, E., Oftebro, R., Laland, S. G., Pettersen, E. O. & Lindmo, T. (1979) Cell-cycle inhibitory effects of the mitotic inhibitor NY 3170 on human cells *in vitro*. *Br. J. Cancer*, **39**, 391.